# Zhinao Capsule improves learning and memory impairment in APP/PS1 mice through gut–brain axis-mediated inhibition of neuroinflammation

**DOI:** 10.3389/fmicb.2026.1735765

**Published:** 2026-02-02

**Authors:** Shuzhen Fang, Hu Xi, Kangyi Zhang, Xiang Fang, Yulong Yang, Jing Li, Wenming Yang

**Affiliations:** 1 First Affiliated Hospital of Anhui University of Traditional Chinese Medicine, Hefei, China; 2 Center for Xin’an Medicine and Modernization of Traditional Chinese Medicine, Institute of Health and Medicine Hefei Comprehensive National Science Center, Hefei, China; 3 Key Laboratory of Xin’an Medicine, Ministry of Education, Hefei, China; 4 Wangjing Hospital, China Academy of Chinese Medical Sciences, Beijing, China; 5 National Key Laboratory for Tea Plant Germplasm Innovation and Resource Utilization, Anhui Agricultural University, Hefei, China; 6 The Second Affiliated Hospital of Anhui University of Chinese Medicine, Hefeis, China

**Keywords:** Alzheimer’s disease, gut microbiota, gut-brain axis, learning and memory ability, neurological function, Zhinao Capsule (ZNJN)

## Abstract

Traditional Chinese Medicine (TCM) interventions have attracted increasing attention in recent years, with a growing body of evidence supporting their efficacy in the treatment of Alzheimer’s disease (AD). Zhinao Capsule (ZNJN), a proprietary TCM formulation, has demonstrated promising clinical outcomes, particularly in enhancing cognitive function and alleviating AD-related pathology in rodent models. This study aimed to evaluate the neuroprotective effects of ZNJN in APP/PS1 transgenic mice. Behavioral assessments indicated that ZNJN, especially at the high dose, significantly improved learning and memory abilities. Histopathological analysis revealed a marked reduction in hippocampal Aβ_1–42_ deposition and decreased activation of microglia and astrocytes, as evidenced by lower expression levels of Iba-1 and GFAP. In addition to central effects, ZNJN alleviated colonic inflammation and improved mucosal integrity. Systemic inflammatory responses were also suppressed, with significant reductions in serum levels of TNF-α, IL-6, IL-1β, and LPS. Furthermore, 16S rRNA gene sequencing showed that ZNJN modulated the gut microbiota by decreasing the abundance of pro-inflammatory genera and enriching potentially beneficial. These findings suggest that ZNJN exerts neuroprotective effects by modulating the gut microbiota and reducing neuroinflammation through the gut–brain axis. These findings suggest that ZNJN exerts neuroprotective effects by modulating the gut microbiota and reducing neuroinflammation through the gut–brain axis. This study provides experimental evidence supporting the potential of ZNJN as a multi-target therapeutic agent for AD intervention.

## 1 Introduction

Alzheimer’s disease (AD), often referred to as senile dementia, is a progressive neurodegenerative disorder characterized by the degeneration and death of neurons. Clinically, it manifests as memory impairment, executive and visuospatial dysfunction, agnosia, aphasia, and notable changes in behavior and personality (Liu et al., 2023). AD predominantly affects individuals aged 65 and older. According to epidemiological studies, it ranks as the fourth leading cause of death globally, following heart disease, cancer, and stroke (Safiri et al., 2024). With the rapid aging of the global population, the prevalence of AD is steadily increasing. Data from the World Health Organization showed that approximately 29.8 million individuals were living with AD in 2015, a number projected to rise to 66 million by 2030 and exceed 115 million by 2050 (Mancuso and Santangelo, 2018; Saddiki et al., 2020). Age remains the most significant risk factor for AD. It was reported that the prevalence of AD is around 5% among individuals aged 65 and older, increasing to 25–30% in those over 85 (Li et al., 2017). These alarming trends highlight the urgent need to unravel the pathogenesis of AD and develop effective therapeutic strategies.

AD is characterized by multifactorial and complex pathological processes, including β-amyloid (Aβ) accumulation, chronic neuroinflammation, glial cell activation, and disturbances in gut microbiota. Among these, the deposition of Aβ, particularly Aβ_1–42_, is considered a central hallmark, due to its strong aggregation tendency and neurotoxicity. These aggregates contribute to neuronal damage and the formation of amyloid plaques. The accumulation of Aβ also triggers the activation of glial cells in the central nervous system (CNS), particularly microglia and astrocytes, marked by ionized calcium-binding adaptor molecule 1 (Iba-1) and glial fibrillary acidic protein (GFAP), respectively. Their activation leads to the release of proinflammatory cytokines such as tumor necrosis factor-alpha (TNF-α), interleukin-1β (IL-1β), and interleukin-6 (IL-6), which sustain a chronic neuroinflammatory environment, exacerbating neuronal degeneration and cognitive decline (Calsolaro and Edison, 2016; Leng and Edison, 2021). Recent studies have highlighted the role of the gut microbiota in AD pathogenesis via the gut–brain axis (GBA). Dysbiosis, or imbalance in gut microbiota, compromises intestinal mucosal integrity, allowing endotoxins such as lipopolysaccharide (LPS) to enter circulation and trigger systemic low-grade inflammation (Huang et al., 2025). Furthermore, microbial metabolites can influence central nervous system inflammation and Aβ metabolism through neuroimmune and neuroendocrine pathways, perpetuating a harmful cycle of microbiota imbalance, inflammation, and neurodegeneration (Bano et al., 2024; Jamerlan et al., 2025). Therefore, therapeutic strategies targeting the gut microbiota, neuroinflammation, and glial cell activation represent promising avenues for AD intervention (Cryan et al., 2020).

In recent years, Traditional Chinese Medicine (TCM) has emerged as a valuable resource for multi-target approaches to complex diseases like AD. Increasing evidence supports the efficacy of TCM-based therapies in improving cognitive function and modulating AD-related pathological mechanisms. Drawing from over two decades of clinical experience, our research group has proposed that “spleen and kidney deficiency” underlies the fundamental pathogenesis of AD, while “phlegm and blood stasis” are the core contributing mechanisms. Based on these principles, we developed a proprietary TCM formula, Zhinao Capsule (ZNJN), composed of eight medicinal ingredients including *Codonopsis pilosula*, *Astragalus membranaceus*, *Polygonatum sibiricum*, *Acorus tatarinowii*, *Cistanche deserticola*, *Curcuma longa*, *Ligusticum chuanxiong*, and Earthworm. Preclinical studies have demonstrated that ZNJN significantly improves learning and memory and alleviates pathological changes in AD rodent models (Han et al., 2003; Ma et al., 2022). However, the precise pharmacological mechanisms underlying the therapeutic effects of ZNJN remain unclear. In particular, the role of the gut microbiota in mediating the neuroprotective effects of ZNJN has yet to be fully elucidated. Given the growing evidence linking gut dysbiosis and AD progression via the gut–brain axis, it is critical to investigate whether ZNJN exerts its effects through modulation of gut microbial composition and related inflammatory pathways.

The present study aims to evaluate the therapeutic potential of ZNJN in APP/PS1 transgenic mice, focusing on its effects on gut microbiota composition, central and peripheral inflammation, Aβ deposition, and glial activation. Specifically, we seek to explore the neuroprotective mechanisms of ZNJN from the perspective of the gut–brain axis. This investigation is expected to provide experimental evidence supporting ZNJN as a multi-target candidate for AD treatment and contribute to a deeper understanding of the therapeutic mechanisms of TCM in neurodegenerative diseases.

## 2 Materials and methods

### 2.1 Materials and experimental animals

Zhinao Capsule (ZNJN, 0.4 g per capsule) was provided by the First Affiliated Hospital of Anhui University of Traditional Chinese Medicine (Batch No.: 20221129). The formulation contains the following primary herbal ingredients: *C. pilosula*, *A. membranaceus*, *P. sibiricum*, *C. deserticola*, *C. longa*, *A. tatarinowii*, *L. chuanxiong*, and *Lumbricus nativus*. ZNJN is an in-house preparation that has been widely applied in clinical practice for over 20 years. Previous studies by our research team have identified its key bioactive constituents as β-sitosterol, quercetin, and baicalein (Ma et al., 2022). Donepezil hydrochloride tablets (5 mg/tablet; Batch No.: 0000018324) were manufactured by Zhejiang Huahai Pharmaceutical Co., Ltd. and used as a positive control. Male APP/PS1 double-transgenic mice (AD model) and age-matched wild-type C57BL/6J mice (6 months old) were purchased from Hangzhou Ziyuan Experimental Animal Technology Co., Ltd. (License No.: 20210516Abbb05000259). All animals were maintained under specific pathogen-free (SPF) conditions with *ad libitum* access to food and water throughout the experimental period. All experimental procedures were conducted in accordance with the institutional guidelines for animal care and approved by the Animal Ethics Committee of Anhui Agricultural University (Approval No.: AHAUB2023024).

### 2.2 Animal experiment and drug intervention

After a 7-day acclimatization period, forty APP/PS1 transgenic mice were randomly assigned into five groups (*n* = 8 per group) including high-dose ZNJN (ZNJN-H), medium-dose ZNJN (ZNJN-M), low-dose ZNJN (ZNJN-L), positive control, and model group. In addition, eight age-matched C57BL/6J mice were assigned to the normal control (Ctrl) group. Mice in both the control and model groups received physiological saline at a dose of 10 mL/kg/day via oral gavage. The ZNJN-L, ZNJN-M, and ZNJN-H groups were administered ZNJN at doses of 0.234, 0.468, and 0.936 g/kg/day, respectively. Mice in the positive control group received donepezil hydrochloride at a dose of 0.65 mg/kg/day. All treatments were administered once daily by oral gavage for a duration of 28 consecutive days.

### 2.3 Behavioral analysis

#### 2.3.1 Barnes Maze test

The Barnes Maze (BM) test was used to evaluate spatial learning and memory in mice. The apparatus consisted of a white, circular platform with a diameter of 120 cm, featuring 20 evenly spaced circular holes (each 10 cm in diameter) positioned around its periphery. Following the training phase, short-term and long-term memory assessments were conducted on days 5 and 12, respectively. During these probe trials, the escape box was removed, allowing the mice to freely explore the maze for up to 90 s in search of the target hole. If a mouse failed to locate the target hole within this time frame, an escape latency of 90 s was recorded. Escape latency, the time taken for a mouse to identify the target hole or enter the escape box, was used as the primary indicator of learning and memory performance, with shorter latencies reflecting better cognitive function. Mouse trajectories were recorded and analyzed using EthoVision XT 14.1 video tracking software.

#### 2.3.2 Two-trial recognition Y-maze

The Y-maze apparatus consisted of three identical arms arranged at 120° angles to form a Y-shape. Each arm measured 30 cm in length, 8 cm in width, and 15 cm in height, and was equipped with a removable barrier at the distal end to allow configuration changes between the training and testing phases. Following a 120-min intertrial interval after the training phase, the testing phase commenced by removing the barrier from the novel arm, thereby allowing the mice to explore all three arms freely for 5 min. Arm entries were recorded using video tracking, and the percentage of entries into the novel arm was calculated. The proportion of novel arm entries served as an index of spatial exploration and recognition memory, with a higher percentage indicating superior cognitive performance.

### 2.4 Sample collection and tissue preparation

Following the final behavioral assessments, mice were fasted for 12 h. Anesthesia was induced by intraperitoneal injection of 2% sodium pentobarbital at a dose of 30 mg/kg. Mice were executed by CO_2_ inhalation anesthesia in batches, and serum, tissue and fecal samples were collected in time for subsequent testing.

### 2.5 Inflammatory cytokine detection in serum

Serum levels of pro-inflammatory cytokines, including TNF-α, IL-1β, IL-6, and LPS, were measured using commercially available enzyme-linked immunosorbent assay (ELISA) kits (Nanjing Jiancheng Bioengineering Institute), following the manufacturers’ protocols.

### 2.6 Histological and immunohistochemical analysis

For histopathological evaluation, hematoxylin, and eosin (H&E) staining was performed on the hippocampal CA1 region and colon tissues to observe neuronal morphology and intestinal structure, respectively. The experiment with three mice per group (*n* = 3) was the histological assessment. Immunohistochemistry (IHC) was used to detect the expression of β-amyloid peptide (Aβ_1–42_) in the brain tissues of each group, as histological observations. In addition, immunofluorescence staining was conducted to assess the expression levels of GFAP and Iba-1, which are markers of astrocyte and microglial activation, respectively.

### 2.7 Gut microbiota analysis

Fresh fecal samples were collected and stored at –80 °C for microbiota analysis. Genomic DNA was extracted using a commercial DNA extraction kit. The bacterial 16S rRNA gene V3–V4 hypervariable regions were amplified by PCR and sequenced using high-throughput sequencing technology. Bioinformatics analysis was performed to evaluate the gut microbial community structure. This included operational taxonomic unit (OTU) clustering, alpha and beta diversity analysis, community composition analysis (including Venn diagrams and taxonomic classification at the genus level), and intergroup difference analysis. Linear discriminant analysis effect size (LEfSe) was used to identify taxa with significant differential abundance between groups. All sequencing and data analysis procedures were carried out by Genesky Biotechnologies Inc. (Shanghai, China).

### 2.8 Statistical analysis

All data are presented as mean ± standard error of the mean (SEM). Statistical analyses were performed using GraphPad Prism version 10. One-way analysis of variance (ANOVA) followed by Tukey’s honestly significant difference (HSD) *post-hoc* test was used to assess differences among groups. For microbial community analysis, LEfSe was employed to identify differentially abundant OTUs between groups. A *p*-value of less than 0.05 was considered statistically significant.

## 3 Results

### 3.1 Effects of ZNJN on spatial learning and memory in APP/PS1 mice

**FIGURE 1:**
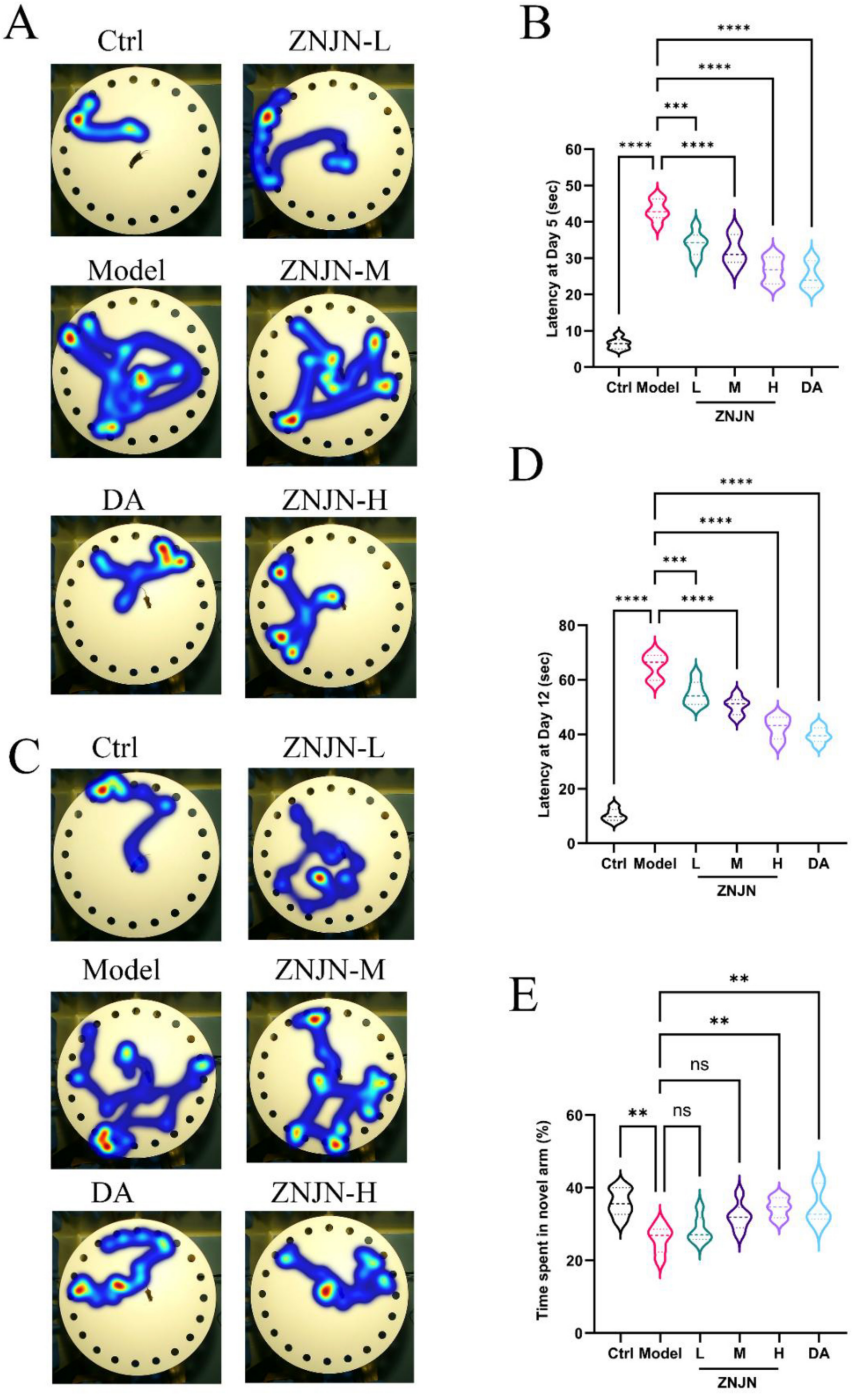
ZNJN improve cognitive impairment of APP/PS1 mice. Barnes maze representing the effects of ZNJN on movement law at day 5 **(A)** and at day 12 **(C)** and latency time to enter the escape box during the short term at day 5 **(B)** and long term at day 12 **(D)** memory test session, respectively. **(E)** Y maze representing the number of each arm entries. The data were represented as mean ± SEM (*n* = 6/group). **p < 0.01, ***p < 0.001,****p < 0.0001.

During the experimental period, all groups of mice underwent Barnes Maze training from days 1 to 4. Short-term and long-term memory were assessed on days 5 and 12, respectively. As shown in Figure 1A, representative movement trajectories demonstrated that Ctrl mice exhibited directed and efficient search paths toward the target escape hole (TEH), indicating intact spatial memory. In contrast, APP/PS1 model mice displayed disorganized and erratic movement patterns, including frequent edge-circling, random hole exploration, and repeated returns to the maze center, reflecting impaired spatial memory and a reliance on non-goal-directed search strategies. Following ZNJN administration, mice exhibited more purposeful and directed trajectories, indicating a clear improvement in cognitive function compared to the untreated model group. In the short-term memory probe trial conducted on day 5 (Figure 1B), Ctrl mice located the TEH in an average of 6.48 ± 1.51 s, whereas the model group required significantly more time, averaging 43.23 ± 2.94 s (*p* < 0.01). ZNJN treatment resulted in a dose-dependent reduction in escape latency. Mice in the low-dose (ZNJN-L), medium-dose (ZNJN-M), and high-dose (ZNJN-H) groups required 33.98 ± 3.33, 32.10 ± 4.15, and 26.73 ± 3.45 s, respectively, to locate the TEH. Notably, the performance of the ZNJN-H group approached that of the positive control group treated with donepezil (DA), which exhibited an escape latency of 25.12 ± 3.68 s.

Similarly, in the long-term memory test conducted on day 12 (Figures 1C,D), Ctrl mice demonstrated a latency of 10.33 ± 2.31 s. The model group showed a marked impairment, taking 65.18 ± 4.52 s to locate the TEH (*p* < 0.01). ZNJN treatment again significantly improved performance in a dose-dependent manner, with the ZNJN-L, ZNJN-M, and ZNJN-H groups requiring 55.02 ± 4.36, 50.50 ± 3.25, and 42.47 ± 4.13 seconds, respectively. The DA group showed the shortest latency among treated groups, at 39.75 ± 2.88 s, comparable to the Ctrl group. In addition to the Barnes Maze test, spatial working memory was assessed using the Y-maze test (Figure 1E). The percentage of entries into the novel arm was 35.90 ± 3.71% in the Ctrl group, indicating normal exploratory behavior. This proportion was significantly reduced in the model group, which showed only 25.90 ± 3.78% novel arm entries (*p* < 0.01), reflecting impaired working memory. Following ZNJN treatment, the proportion of novel arm entries increased progressively with dosage. Specifically, the ZNJN-L group showed 28.47 ± 3.65%, the ZNJN-M group 32.09 ± 3.78%, and the ZNJN-H group 34.55 ± 2.75%. The DA group exhibited a novel arm entry proportion of 35.12 ± 5.35%, closely resembling that of the Ctrl group. The improvement observed in the ZNJN-H group was statistically significant compared to the model group (*p* < 0.01), indicating a substantial enhancement of spatial working memory. Thus, these findings demonstrate that ZNJN significantly ameliorated cognitive deficits in APP/PS1 mice. The effect was dose-dependent, with the high-dose group showing the greatest improvement across both behavioral paradigms. These results suggest that ZNJN has the potential to improve both spatial learning and memory retention in Alzheimer’s disease model mice.

### 3.2 Effects of ZNJN on Aβ_1–42_ deposition and CA1 neuronal morphology in the hippocampus of APP/PS1 mice

**FIGURE 2:**
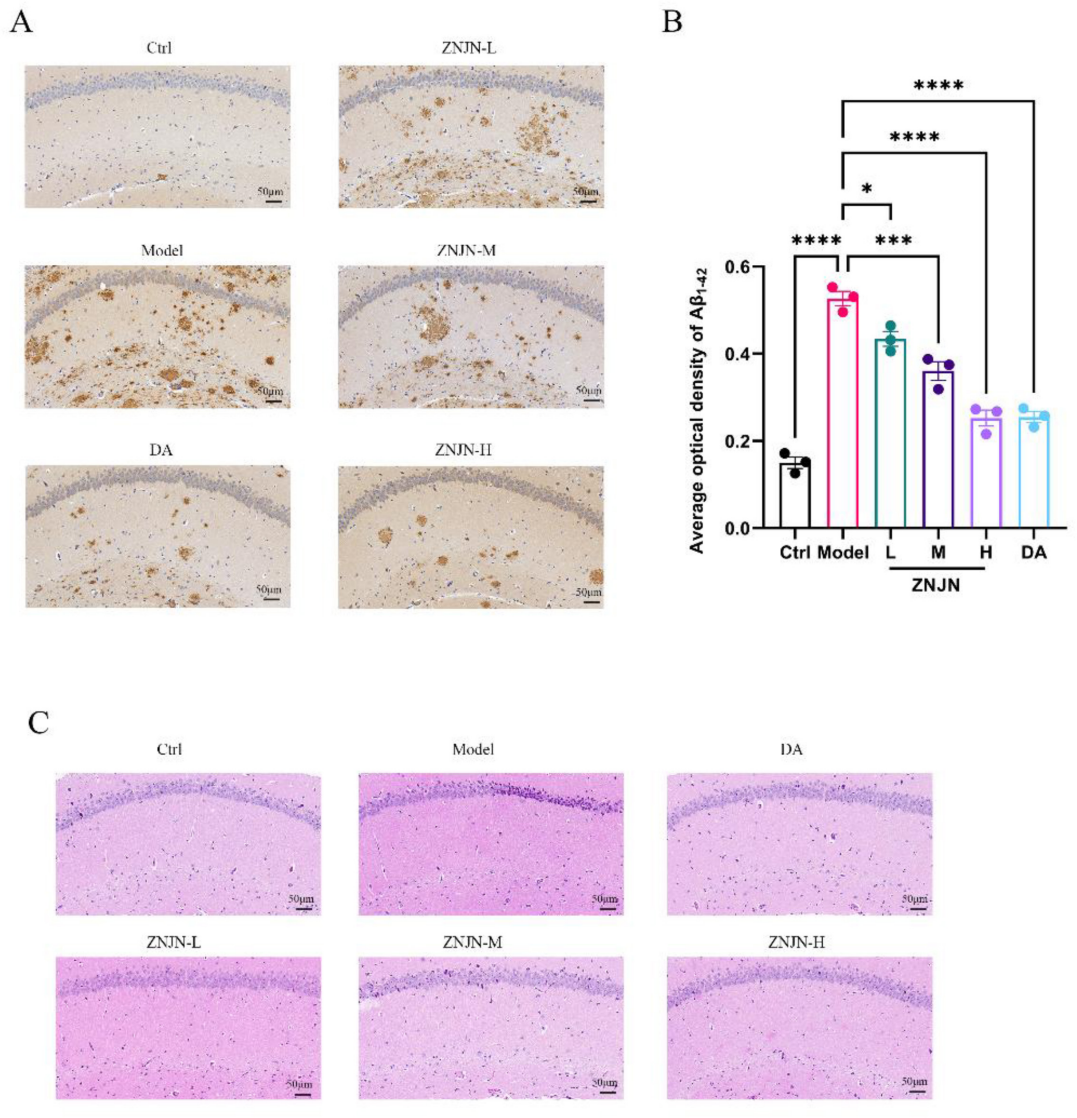
ZNJN on Aβ_1–42_, and CA1 neuronal morphology in the brain tissue of APP/PS1 mice. **(A)** Immunohistochemical analysis for Aβ_1–42_. **(B)** Relative quantification of fluorescence intensity. **(C)** CA1 neuronal morphology. The data were represented as mean ± SEM (*n* = 3/group). *p < 0.05, ***p < 0.001, ****p < 0.0001.

Aβ_1–42_ is a key pathological hallmark in the development of AD, with its abnormal aggregation and deposition in the brain closely associated with disease progression. As a highly amyloidogenic isoform of the Aβ peptide, Aβ_1–42_ tends to form insoluble plaques that contribute to synaptic dysfunction, neuronal loss, and cognitive decline. Its elevated levels in cerebrospinal fluid or plasma have been widely recognized as a diagnostic biomarker and an indicator of disease severity (Min-Kaung-Wint-Mon et al., 2024). As shown in Figure 2A, immunohistochemical analysis revealed distinct Aβ_1–42_-positive plaques in the brain tissues of APP/PS1 model mice. In particular, the model group exhibited strong immunoreactivity, with extensive localized aggregation of Aβ_1–42_ throughout the hippocampal and cortical regions. In contrast, treatment with ZNJN markedly attenuated Aβ_1–42_ deposition in a dose-dependent manner. ZNJN-H and DA groups showed the most pronounced reductions in plaque burden, with smaller, less dense deposits and weaker staining intensity. Quantitative analysis of average optical density (Figure 2B) confirmed a significant increase in Aβ_1–42_ deposition in the model group compared to the Ctrl group (*p* < 0.01). Post-treatment analysis demonstrated a significant decrease in Aβ_1–42_ levels in all treatment groups, with the ZNJN-H and DA groups exhibiting reductions that were statistically significant relative to the model group (*p* < 0.01).

In addition to Aβ pathology, neuronal morphology in the hippocampal CA1 region was evaluated using H&E staining (Figure 2C). In the Ctrl group, CA1 neurons were large, round, and evenly distributed, arranged in tightly packed layers with well-preserved cytoplasmic and nuclear structures. Conversely, neurons in the model group exhibited severe structural damage, including nuclear condensation, cytoplasmic shrinkage, irregular cell contours, and disorganized alignment, indicative of neurodegeneration. Following treatment, neuronal morphology showed marked improvement across all ZNJN and DA groups. In the ZNJN-H and DA groups, neurons appeared more uniform in shape and size, with intact nuclei and reduced intercellular spacing, closely resembling the normal architecture observed in Ctrl mice. The ZNJN-M group also demonstrated moderate improvement, with clearer stratification and reduced morphological abnormalities. However, in the ZNJN-L group, although some neuroprotective effects were evident, neurons remained loosely arranged with noticeable nuclear swelling and increased inter-neuronal gaps, suggesting only partial recovery. Collectively, these findings indicate that ZNJN exerts neuroprotective effects by reducing Aβ_1–42_ burden and preserving hippocampal neuronal structure, with the high-dose treatment showing efficacy comparable to the positive control (DA). These effects are consistent with the observed improvements in cognitive function described in the behavioral assessments.

### 3.3 Effects of ZNJN on hippocampal expression of Iba-1 and GFAP in APP/PS1 mice

**FIGURE 3:**
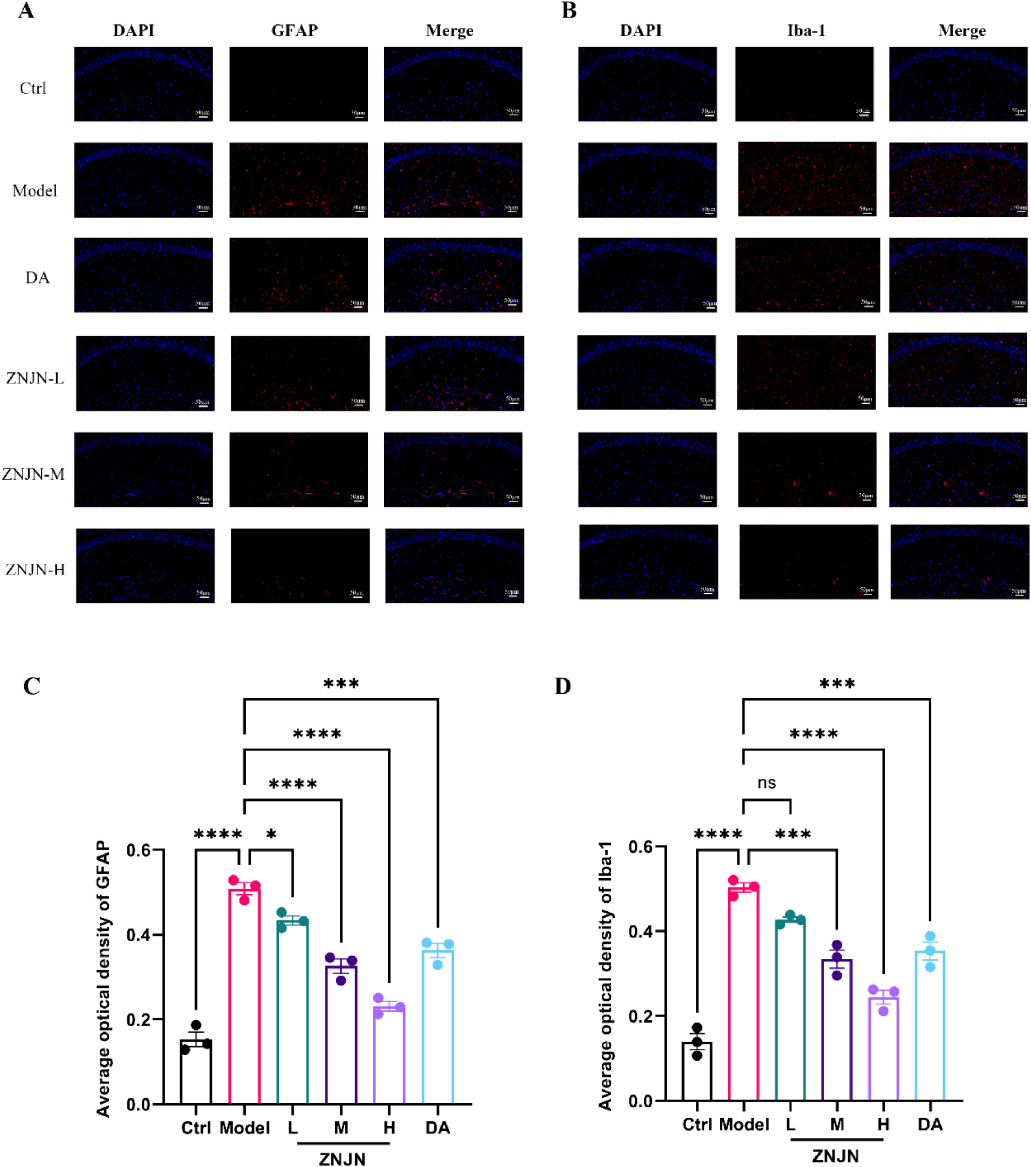
Influence of ZNJN on GFAP, Iba-1 expression in the brain tissue of APP/PS1 mice. Fluorescence images of GFAP **(A)** and Iba-1 **(B)** in the brain tissue of APP/PS1 mice. Quantitative analysis of GFAP **(C)** and Iba-1 **(D)** fluorescence content. The data were represented as mean ± SEM (*n* = 3/group). *p < 0.05, ***p < 0.001, ****p < 0.0001.

GFAP and Iba-1 are widely recognized as key markers of astrocyte and microglial activation, respectively. In the central nervous system, GFAP is predominantly expressed in astrocytes, where it contributes to the maintenance of neuronal structural integrity and glial homeostasis. In contrast, Iba-1 is specifically expressed in microglia and is markedly upregulated in response to neuroinflammatory stimuli, injury, or neurodegenerative processes. As illustrated in Figure 3A, hippocampal GFAP expression was significantly elevated in the APP/PS1 model group compared to Ctrl group, indicating robust astrocyte activation. Following ZNJN treatment, GFAP expression levels declined across all intervention groups. This reduction was most pronounced in ZNJN-H and DA groups, both of which showed significantly lower GFAP immunoreactivity relative to the model group (*p* < 0.01) (Figure 3C).

Similarly, immunofluorescence analysis of Iba-1 expression revealed marked microglial activation in the model group (Figure 3B). Quantitative assessment of average optical density values (Figure 3D) confirmed a significant upregulation of Iba-1 in the model group compared to the Ctrl group (*p* < 0.01). ZNJN administration led to a dose-dependent reduction in Iba-1 expression, with the ZNJN-H and DA groups again exhibiting the most substantial decreases (*p* < 0.01). Collectively, these findings suggest that ZNJN effectively attenuates glial activation in the hippocampus of APP/PS1 mice, as evidenced by the downregulation of GFAP and Iba-1 expression. This implies a potential role for ZNJN in the suppression of neuroinflammation and the preservation of neuronal homeostasis in Alzheimer’s disease pathology.

### 3.4 Effects of ZNJN on colonic morphology in APP/PS1 mice

**FIGURE 4:**
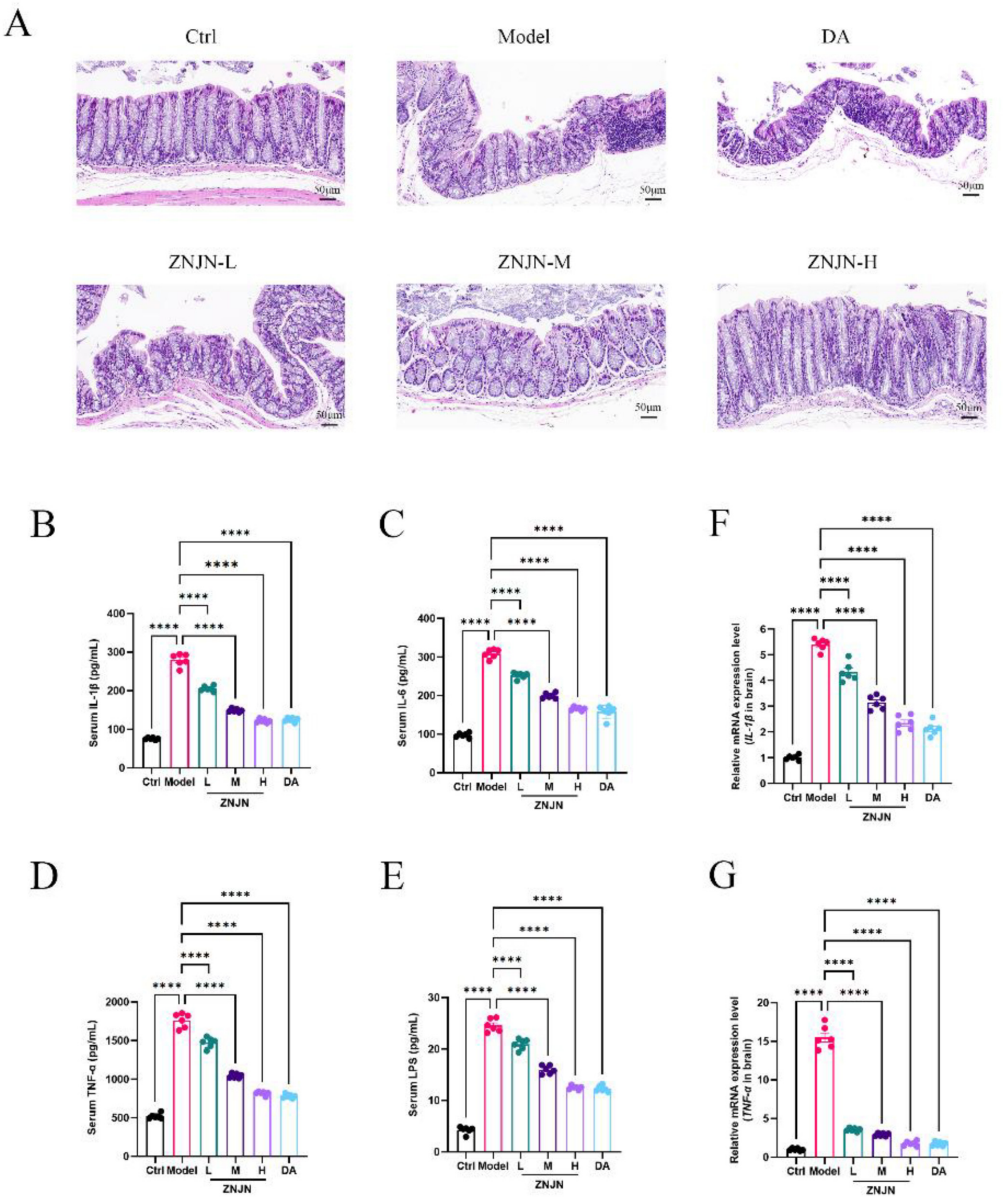
Influence of colon morphology and inflammatory reaction in the APP/PS1 mice. **(A)** HE staining of colon tissue. **(B–E)** Serum levels of IL-1β, IL-6, TNF-α and LPS. **(F,G)** mRNA expression levels of *IL-1*β and *TNF-*α. The data were represented as mean ± SEM (*n* = 6/group). ****p < 0.001.

H&E staining was performed to evaluate colonic tissue morphology across the different experimental groups, as shown in Figure 4A. In Ctrl group, the colonic mucosa exhibited intact architecture, characterized by well-organized and uniformly distributed villi. No signs of edema, vascular congestion, or inflammatory cell infiltration were observed, indicating normal intestinal integrity. In contrast, colonic tissues from the APP/PS1 model group displayed pathological alterations indicative of mild inflammation. These included mucosal edema, infiltration of inflammatory cells into the epithelial and lamina propria layers, and disruption of villus architecture. The mucosal surface appeared irregular, with shortened, uneven, and partially detached villi.

ZNJN treatment resulted in a dose-dependent improvement in colonic morphology. In the ZNJN-L, ZNJN-M, and ZNJN-H groups, reductions in mucosal edema and inflammatory infiltration were observed, along with partial restoration of villus structure. Although mild epithelial shedding persisted in some cases, the overall mucosal architecture was markedly more preserved compared to the model group. Among the treatment groups, the ZNJN-H group exhibited the most substantial histological improvement, with relatively intact and orderly villi, suggesting a strong protective effect on the intestinal barrier. Interestingly, DA group showed minimal morphological improvement relative to the model group, indicating that ZNJN may have unique protective effects on intestinal structure that are not shared by standard anti-AD pharmacotherapy. These findings suggest that ZNJN not only exerts neuroprotective effects but may also contribute to the maintenance of intestinal mucosal integrity, potentially through modulation of systemic inflammation or the gut–brain axis.

### 3.5 Influence of ZNJN on inflammatory mediators in serum of mice

As shown in Figures 4B–E, modeled mice demonstrated substantial increase TNF-α, IL-6, IL-1β, and LPS (*p* < 0.01) levels in serum compared with Ctrl. After ZNJN intervention, TNF-α, IL-6, IL-1β, and LPS (*p* < 0.01) levels in serum were markedly lowered in all treatment groups in comparison with the model (*p* < 0.01). Among the treatments, the ZNJN-H and DA groups showed the most pronounced effects, which was statistically significant (*p* < 0.01). The results indicate that ZNJN, particularly at higher doses, can effectively reduce inflammation markers in AD mice, highlighting their potential anti-inflammatory effects in AD.

### 3.6 Effects of ZNJN on systemic inflammatory mediators in the serum of APP/PS1 mice

As shown in Figures 4B–E, serum levels of pro-inflammatory mediators, including TNF-α, IL-6, IL-1β, and LPS, were significantly elevated in the APP/PS1 model group compared to Ctrl group (*p* < 0.01), indicating a pronounced systemic inflammatory response associated with Alzheimer’s disease pathology. Following ZNJN treatment, all dosage groups exhibited marked reductions in serum concentrations of TNF-α, IL-6, IL-1β, and LPS when compared to the model group (*p* < 0.01). Notably, ZNJN-H and DA groups demonstrated the most substantial decreases in these inflammatory markers, with differences reaching statistical significance relative to the model group (*p* < 0.01). These findings indicate that ZNJN possesses significant anti-inflammatory properties, particularly at higher doses. By effectively suppressing systemic inflammatory mediators, ZNJN may contribute to the mitigation of neuroinflammation and associated pathological processes in Alzheimer’s disease.

### 3.7 Effects of ZNJN on mRNA Expression of inflammatory cytokines in Hippocampus tissue of APP/PS1 mice

RT-qPCR analysis of inflammatory cytokine mRNA expression levels in mice hippocampal tissue (as shown in Figures 4F,G) revealed significantly elevated IL-1β and TNF-α mRNA expression in the Model group compared to the Control group (*p* < 0.05). Following drug treatment, mRNA expression levels of IL-1β and TNF-α in hippocampal tissue were significantly reduced in treated mice, with more pronounced effects observed in the ZNJN-H and DA groups (*p* < 0.05).

### 3.8 Effects of ZNJN on gut microbiota composition and diversity in APP/PS1 mice

**FIGURE 5:**
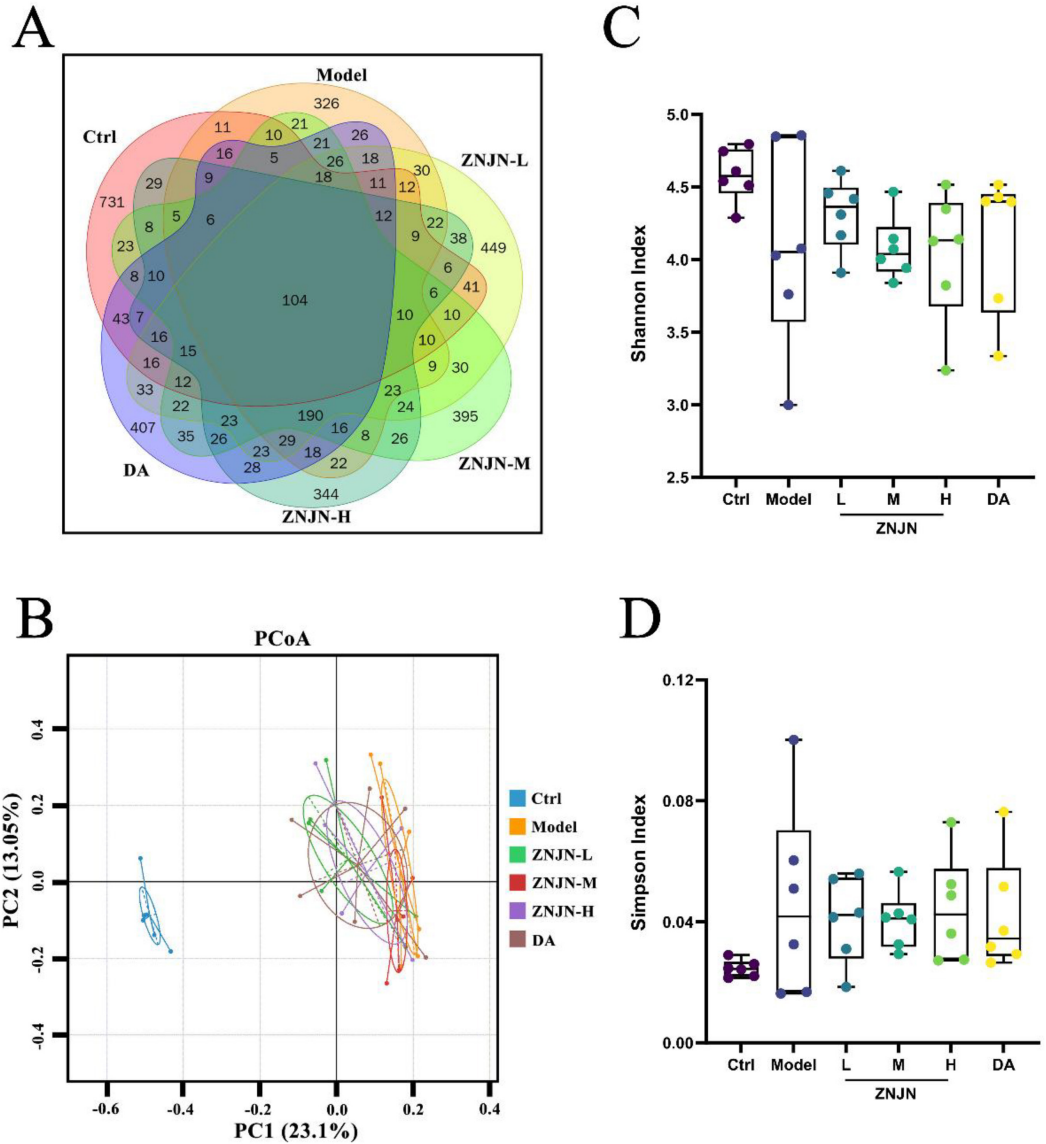
Structure and composition of gut microbiota of mice. **(A)** Venn diagram, **(B)** PCoA, **(C)** Shannon, and **(D)** Simpson.

To investigate the impact of ZNJN on gut microbiota, a comprehensive analysis of microbial diversity and taxa composition was conducted. As shown in Figure 5A, a Venn diagram illustrating OTUs revealed that all six experimental groups shared 104 OTUs. However, Ctrl group exhibited a total of 731 OTUs, whereas the APP/PS1 model group displayed a marked reduction to 326 OTUs, indicating a significant loss of microbial diversity associated with Alzheimer’s disease pathology. PCoA was subsequently performed to assess β-diversity among groups (Figure 5B). The first two principal components (PC1 and PC2) accounted for 23.1 and 13.05% of total variance, respectively. The model group was clearly separated from the Ctrl group, reflecting substantial microbial dysbiosis. Notably, the microbiota profiles of ZNJN-treated mice, particularly ZNJN-H group, shifted closer to the Ctrl group, suggesting partial restoration of microbial community structure following treatment. To evaluate α-diversity, Shannon and Simpson indices were calculated (Figures 5C,D). The model group exhibited a significant reduction in the Shannon index, indicative of decreased microbial diversity. ZNJN administration led to an upward trend in Shannon index values across all treated groups, although the differences were not statistically significant. The Simpson index demonstrated a corresponding decrease following ZNJN intervention, consistent with improved community evenness; however, this change also lacked statistical significance. Overall, these data suggest that ZNJN treatment contributed to microbial diversity in APP/PS1 mice.

**FIGURE 6:**
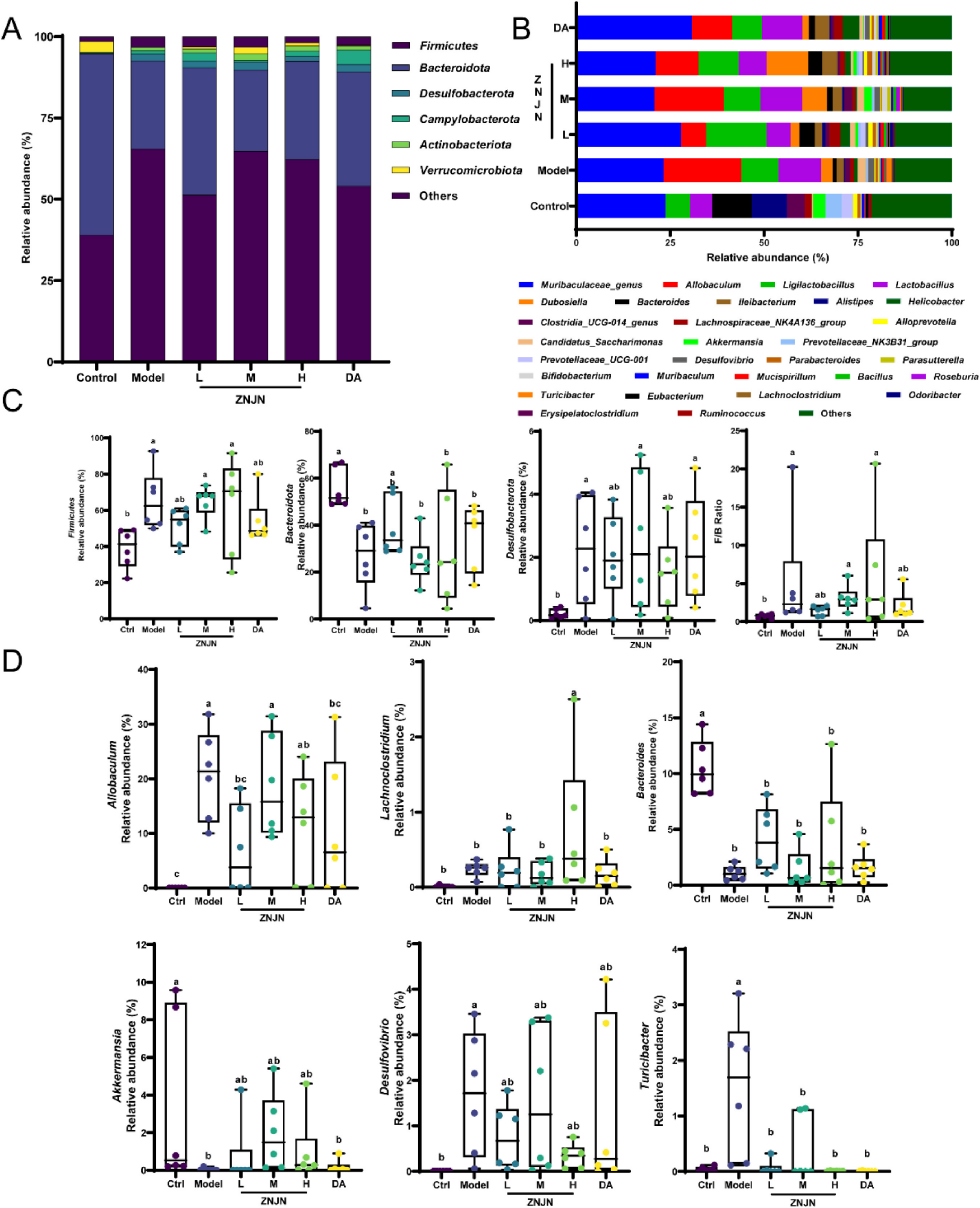
Composition and relative abundance of gut microbiota of mice. Stacked horizontal bar chart showing the relative abundance at the phylum level **(A)** and at the genus level **(B)**. **(C)** Relative abundance of *Firmicutes*, *Bacteroidota*, and *Desulfobacterota*, along with the Firmicutes/Bacteroidota (F/B) ratio. **(D)** Relative abundance of selected genera: *Allobaculum*, *Lachnoclostridium*, *Bacteroides*, *Akkermansia*, *Desulfovibrio*, and *Turicibacter*.

At the phylum level (Figures 6A,C), the relative abundance of Bacteroidetes was significantly reduced in the model group compared to the Ctrl (*p* < 0.01), while Firmicutes and Desulfobacterota were significantly increased, resulting in a notably elevated Firmicutes/Bacteroidetes (F/B) ratio. ZNJN-H treatment partially reversed these changes, increasing the relative abundance of Bacteroidetes and decreasing that of Firmicutes and Desulfobacterota, although the differences were not statistically significant. At the genus level (Figures 6B,D), the gut microbiota was predominantly composed of *Allobaculum*, *Ligilactobacillus*, *Akkermansia*, *Lachnoclostridium*, *Dubosiella*, and *Bacteroides*. Relative to the Ctrl group, the model group showed significant reductions in *Bacteroides* and *Akkermansia* (*p* < 0.01), while *Allobaculum*, *Desulfobacterota*, and *Turicibacter* were markedly elevated (*p* < 0.01). ZNJN-H treatment resulted in a significant increase in the relative abundance of *Lachnoclostridium* (*p* < 0.01), and partial recovery of *Bacteroides* and *Akkermansia*, though these changes were not statistically significant. Additionally, a significant decrease in *Turicibacter* was observed in the ZNJN-H group compared to the model group (*p* < 0.01), indicating a potential reversal of pro-inflammatory microbial shifts.

**FIGURE 7:**
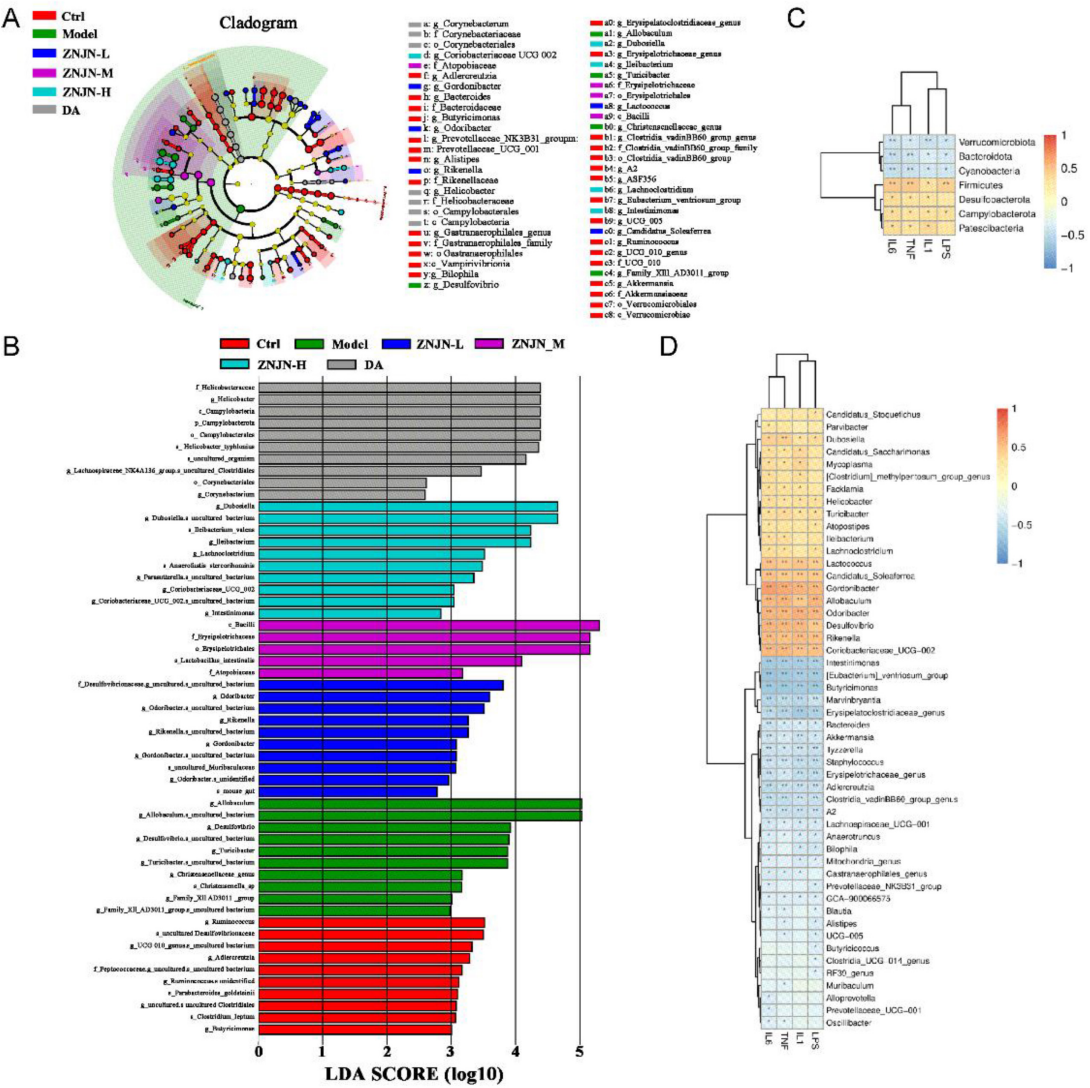
LEfSe analysis and correlation between gut microbiota and serum inflammatory factors. **(A)** LEfSe cladogram indicating differentially abundant taxa among groups. **(B)** LDA score bar plot showing taxa with significant differences in abundance. **(C)** Heatmap showing the correlations between serum inflammatory cytokines (columns) and gut microbiota (rows) at the phylum level. **(D)** Heatmap showing the correlations between serum inflammatory cytokines and gut microbiota at the genus level. Red indicates a positive correlation, and blue indicates a negative correlation.

To identify taxa most influenced by ZNJN treatment, LEfSe was performed on OTUs with relative abundance > 0.5% (Figures 7A,B). In the model group, *Allobaculum*, *Desulfovibrio*, and *Turicibacter* were dominant, whereas the Ctrl group was characterized by *Ruminococcus*, *UCG_010_genus*, and *Adlercreutzia*. The ZNJN-H group showed enrichment in several potentially beneficial genera, including *Dubosiella*, *Ileibacterium*, *Lachnoclostridium*, *Parasutterella*, *Coriobacteriaceae_UCG_002*, and *Intestinimonas*, as well as two specific species, *Anaerofustis stercorihominis* and *Ileibacterium valens*. In contrast, the DA group was dominated by Helicobacter and Lachnospiraceae_NK4A136_group.

### 3.9 Correlation between serum inflammatory mediators and gut microbiota in APP/PS1 mice

To evaluate the relationship between systemic inflammation and gut microbiota, Spearman correlation analysis was conducted, aiming to identify microbial taxa significantly associated with inflammatory mediators. The results are visually presented at both the phylum level (Figure 7C) and genus level (Figure 7D). At the phylum level, Firmicutes, Desulfobacterota, Campylobacterota, and Patescibacteria exhibited significant positive correlations (*p* < 0.01) with pro-inflammatory cytokines TNF-α, IL-1β, and IL-6. Additionally, LPS levels were positively correlated (*p* < 0.01) with Firmicutes and Campylobacterota, but not with Desulfobacterota or Patescibacteria. In contrast, Verrucomicrobiota, Bacteroidota, and Cyanobacteria showed significant negative correlations (*p* < 0.01) with TNF-α, IL-1β, IL-6, and LPS, suggesting a potential anti-inflammatory role for these phyla.

At the genus level, 14 genera demonstrated significant positive correlations with TNF-α, IL-1β, and IL-6 (*p* < 0.01), including *Facklamia*, *Helicobacter*, *Turicibacter*, *Atopostipes*, *Ileibacterium*, *Lachnoclostridium*, *Lactococcus*, *Candidatus_Soleaferrea*, *Gordoni- bacter*, *Allobaculum*, *Odoribacter*, *Desulfovibrio*, *Rikenella*, and *Coriobacteriaceae_UCG-002*. Conversely, multiple genera were negatively correlated (*p* < 0.01) with all four inflammatory mediators including TNF-α, IL-1β, IL-6, and LPS. These included *Intestinimonas*, *[Eubacterium]_ventriosum_group*, *Butyrici- monas*, *Marvinbryantia*, *Erysipelatoclostridiaceae_genus*, *Bacter- oides*, *Akkermansia*, *Tyzzerella*, *Staphylococcus*, *Erysipelo- trichaceae_genus*, *Adlercreutzia*, and *Clostridia_vadinBB60_ group_genus*. These taxa may exert anti-inflammatory effects through gut–immune interactions.

Among the identified genera, a substantial number exhibited statistically significant correlations with the measured inflammatory mediators. Specifically, IL-1β was significantly associated with 34 genera, of which 15 were positively and 19 negatively correlated. Similarly, TNF-α showed significant correlations with 38 genera (16 positive, 22 negative), and IL-6 was linked to 43 genera, including 19 with positive and 24 with negative associations. In the case of LPS, significant correlations were observed with 39 genera, comprising 14 positive and 25 negative relationships.

These results highlight a consistent pattern wherein a greater proportion of microbial genera demonstrated negative correlations with systemic inflammatory markers, suggesting that many of these microbes may play a protective or anti-inflammatory role in the host. Conversely, a subset of genera exhibited positive associations, implying potential involvement in pro-inflammatory pathways. This duality underscores the complexity of hostd positive associations, implying potential involvement disease and supports the hypothesis that modulation of specific microbial taxa may influence systemic inflammation and disease progression.

## 4 Discussion

Emerging evidence highlights the central role of the gut microbiota in the development and progression of AD (Jamerlan et al., 2025). Alterations in microbial composition and diversity have been consistently observed in both clinical populations and AD animal models, implicating the gut microbiota as a key modulator in the pathophysiology of neurodegeneration (Harach et al., 2017; Zhuang et al., 2018). These findings support the increasingly accepted concept of the GBA, in which bidirectional communication between the gastrointestinal tract and the CNS influences neuroinflammatory processes, amyloid deposition, and cognitive function (Chen et al., 2022; Kapoor et al., 2024). As such, targeted modulation of the gut microbiota holds promise as an innovative therapeutic strategy for AD management (Li et al., 2019; Sun et al., 2019). Our previous studies identified quercetin, baicalin, and luteolin as the primary active constituents of ZNJN, a traditional Chinese medicinal formulation. These phytochemicals exhibit strong affinities for AD-related molecular targets and have demonstrated neuroprotective properties through anti-inflammatory and antioxidant mechanisms (Wei et al., 2022). In rodent models, they have been shown to improve memory performance and reduce Aβ deposition, thereby enhancing spatial learning and memory. Building on these findings, the present study investigated whether ZNJN exerts its anti-AD effects through modulation of the gut microbiota and its downstream impact on neuroinflammation and amyloid pathology (Zhang et al., 2024). However, these studies have largely overlooked the potential involvement of the gut microbiota in mediating these neuroprotective effects. To address this gap, the present study focused on characterizing the alterations in gut microbiota composition in AD model mice and investigating whether ZNJN could exert beneficial effects via modulation of the gut microbiome.

Our findings revealed that the APP/PS1 transgenic mice exhibited pronounced gut dysbiosis relative to the control group, as evidenced by a significant reduction in microbial diversity and marked alterations in community composition across multiple taxonomic levels (Shen et al., 2017; Chen et al., 2020). This microbial imbalance was accompanied by elevated serum concentrations of key proinflammatory mediators, including TNF-α, IL-1β, IL-6, and LPS, as well as histological evidence of colonic epithelial damage and disrupted intestinal barrier integrity (Silva et al., 2020; Chen et al., 2024). These results support the growing consensus that alterations in gut microbiota are closely linked to systemic inflammation and immune dysregulation in the context of AD pathogenesis (Cattaneo et al., 2017; Vogt et al., 2017). Importantly, intervention with ZNJN, particularly at higher doses, partially reversed these pathological alterations. ZNJN treatment not only restored microbial diversity but also restructured the microbial community toward a composition more closely resembling that of healthy controls. This microbiota remodeling was paralleled by a significant reduction in circulating inflammatory mediators and improved intestinal histology, suggesting that ZNJN may exert its therapeutic effects, at least in part, through the restoration of gut microbial homeostasis. These findings highlight the potential of ZNJN to modulate host physiology via microbiota-dependent mechanisms, reinforcing the critical role of the gut–brain axis in mediating neuroinflammatory processes and AD-like pathology.

At the phylum level, the gut microbiota of model mice displayed a marked reduction in Bacteroidetes and enrichment of Firmicutes and Desulfobacterota, resulting in an increased F/B ratio, a widely recognized indicator of dysbiosis (Zhu et al., 2024). Elevated F/B ratios have been associated with metabolic disturbances, increased intestinal permeability, and chronic inflammation (Turnbaugh et al., 2006; Meijer et al., 2010). The enrichment of Desulfobacterota, a group of sulfate-reducing bacteria, may further exacerbate mucosal damage through the production of hydrogen sulfide, contributing to oxidative stress and proinflammatory signaling (Carbonero et al., 2012). These changes are consistent with the elevated serum LPS levels observed in model mice, which likely reflect increased translocation of bacterial endotoxins into the bloodstream due to compromised gut barrier integrity. LPS, as a potent ligand of toll-like receptor 4 (TLR4), can activate microglia via the TLR4/NF-κB signaling pathway, thereby stimulating the release of proinflammatory cytokines and promoting neuroinflammation (Chen et al., 2023). ZNJN treatment significantly reduced serum levels of proinflammatory cytokines and LPS, suggesting a dual mechanism of action involving both restoration of intestinal barrier function and suppression of gut-derived inflammation (Martínez-Díaz et al., 2020; Hamed et al., 2024). The observed increase in Bacteroidetes following ZNJN administration is particularly noteworthy, as members of this phylum are key producers of short-chain fatty acids (SCFAs), such as butyrate and propionate, which are known to reinforce epithelial barrier integrity, suppress inflammatory signaling, and inhibit the growth of pathogenic bacteria (Koh et al., 2016; Saad and El-Din, 2024). These findings suggest that ZNJN may mitigate systemic inflammation by restoring SCFA-producing microbial populations and reducing endotoxin burden.

In parallel with these peripheral changes, ZNJN treatment also attenuated central neuroinflammatory responses. Model mice exhibited elevated expression of GFAP and Iba-1 in the hippocampus, indicating activation of astrocytes and microglia, along with increased deposition of Aβ_1–42_. These pathological hallmarks were significantly alleviated following ZNJN intervention, suggesting that gut microbiota modulation may indirectly influence CNS homeostasis via the GBA (Qu et al., 2024). Among the proinflammatory taxa enriched in the model group at genus level, *Desulfovibrio* and *Turicibacter* were of particular interest. *Desulfovibrio*, a known producer of hydrogen sulfide and LPS, may exacerbate gut permeability and systemic inflammation (Zhang et al., 2023; Huang et al., 2024; Singh et al., 2024), while *Turicibacter* has been implicated in disruptions of tryptophan metabolism and reduction of neuroprotective indole derivatives (Price et al., 2024; Golob et al., 2025). The suppression of these taxa by ZNJN may represent a key mechanism through which the compound exerts its neuroprotective effects. Conversely, ZNJN enriched several potentially beneficial, including *Dubosiella*, *Lachnoclostridium*, *Alistipes stercorihominis*, and *Intestinimonas valens*. *Dubosiella* is a major SCFAs producer, particularly of butyrate, which upregulates tight junction proteins, such as occludin and ZO-1, via PPAR-γ signaling, thereby enhancing epithelial integrity and limiting systemic inflammation (Kapoor et al., 2024). *Lachnoclostridium* may convert primary bile acids into secondary bile acids, such as lithocholic acid, which activate the Farnesoid X receptor (FXR) and promote Aβ clearance through upregulation of Aβ-degrading enzymes like insulin-degrading enzyme (IDE) (Bedon et al., 2024). *A. stercorihominis* is known to produce succinate, an anti-inflammatory metabolite that inhibits M1 polarization of microglia, thereby reducing neuroinflammatory damage (Verhaar et al., 2021). In addition, *I. valens* may regulate the Th17/Treg balance in the gut, potentially limiting the peripheral immune infiltration into the CNS (Aaldijk and Vermeiren, 2022). These findings suggest that ZNJN may exert multifaceted neuroprotective effects through a synergistic modulation of microbial metabolites, immune responses, and gut–brain communication.

In our study, DA improved cognitive and pathological features of AD, the improvement in intestinal morphology was limited. This may result from several factors. First, ZNJN is a compound herbal medicine containing multiple bioactive ingredients that may act directly on the gut through anti-inflammatory effects and regulation of the gut microbiome, while DA’s main mechanism is inhibition of acetylcholinesterase in the brain. Second, the relatively short treatment duration (28 days) may not have been sufficient to produce morphological changes in the gut. Indeed, previous studies in APP/PS1 mice have reported that Donepezil reduces colonic inflammation and alters gut microbiota composition (Li Y. et al., 2023). Third, the dose of DA used (0.65 mg/kg) was selected based on its central cognitive effects, and may be below the dose required to confer structural benefits to the intestinal mucosa.

Notably, DA has shown some gut-protective actions in other models: in a rat model of doxorubicin-induced gut injury, DA prevented epithelial disruption and preserved barrier integrity (Suparan et al., 2022). Additionally, in a murine model of ulcerative colitis, oral DA reduced inflammation and apoptosis in the colon via LRP1/AMPK/NF-κB signaling (Li A. et al., 2023). These studies suggest that DA can act on the gut under certain pathological conditions, but its effects may depend heavily on model context, dose, and duration.

Spearman correlation analyses further validated the associations between specific microbial taxa and key inflammatory mediators. Phyla such as Firmicutes, Desulfobacterota, and Campylobacterota exhibited positive correlations with TNF-α, IL-1β, and IL-6, supporting their proinflammatory potential (Wang et al., 2024). In contrast, Verrucomicrobiota and Bacteroidota were negatively associated with these cytokines, highlighting their potential anti-inflammatory roles. At the genus level, *Helicobacter* and *Desulfovibrio* were positively correlated with systemic inflammation, while *Akkermansia* and *Bacteroides* displayed inverse correlations, likely due to their SCFA production and barrier-enhancing effects (Choi and Mook-Jung, 2023; Zhou et al., 2024). These findings support the hypothesis that gut dysbiosis exacerbates neuroinflammation and may directly contribute to AD progression via disruption of the GBA (Li et al., 2024). LEfSe analysis further confirmed that model group mice were enriched with proinflammatory taxa, including *Turicibacter* and *Lachnoclostridium*, while beneficial genera such as *Akkermansia* and *Bacteroides* were significantly depleted (He et al., 2024; Khleborodova et al., 2024). These compositional shifts are consistent with previous findings in AD patients and animal models, and likely contribute to disease progression through multiple mechanisms involving inflammation, SCFAs metabolism, and gut barrier integrity (Gangzheng et al., 2023). But LEfSe does not fully correct for multiple testing and that more rigorous statistical validation would be required in future studies.

Collectively, this study provides compelling evidence that ZNJN mitigates AD-like pathology by remodeling the gut microbiota, restoring gut barrier function, and attenuating systemic and central inflammation. These findings underscore the therapeutic potential of gut microbiota-targeted interventions in neurodegenerative diseases and highlight the importance of understanding host–microbiota interactions in the context of AD pathogenesis. Future studies should further elucidate the functional metabolites and molecular pathways involved, particularly through the application of metabolomics, spatial transcriptomics, and single-cell analyses (Aaldijk and Vermeiren, 2022). Moreover, exploring synergistic effects between ZNJN and conventional therapies like donepezil may offer novel avenues for combination treatments (Boţa et al., 2024). Furthermore, robiotic interventions targeting specific genera such as *Akkermansia* or *Bacteroides* may serve as adjunct strategies to reinforce gut barrier integrity and suppress neuroinflammation in AD. The microbiota results in the present study are descriptive and should be interpreted with caution, as functional roles of the altered taxa were inferred from previously published studies rather than direct experimental validation. Although the results demonstrate promising translational potential through microbiota modulation, the use of a single animal model represents an inherent limitation. Additional validation using alternative disease models and human-based studies will be necessary to confirm the robustness and clinical applicability of these findings.

## 5 Conclusion

In summary, this study provides compelling evidence for a strong association between gut microbiota dysbiosis and the pathological progression of AD. The observed microbial imbalance, characterized by a depletion of health-promoting gut microbiota and an enrichment of proinflammatory taxa, was closely linked to systemic inflammation, intestinal barrier dysfunction, and AD-related neuropathology. Notably, intervention with ZNJN effectively ameliorated key AD-related phenotypes. ZNJN administration reduced hippocampal Aβ accumulation and neuroinflammation, improved intestinal barrier integrity, and lowered peripheral levels of TNF-α, IL-1β, IL-6, and LPS. These beneficial effects were closely associated with the restoration of microbial diversity and composition. Specifically, ZNJN enriched beneficial microbes such as *Dubosiella*, *A. stercorihominis*, and *Lachnoclostridium*, which are known to produce anti-inflammatory or neuroprotective metabolites, while suppressing the abundance of proinflammatory bacteria such as *Desulfovibrio* and *Turicibacter*. These findings highlight that ZNJN not only mitigates neuropathological hallmarks of AD but also exerts systemic anti-inflammatory effects through microbiota-dependent mechanisms. The gut microbiota–inflammation axis thus emerges as a critical therapeutic target in AD, with ZNJN representing a promising microbiota-modulating intervention. Nevertheless, further mechanistic studies and clinical validations are needed to clarify causality, identify key microbial metabolites, and assess long-term efficacy and safety. As spleen–kidney deficiency biomarkers were not measured, future studies are needed to integrate TCM syndrome indicators with gut–brain axis mechanisms.

## Data Availability

The original contributions presented in this study are included in this article/supplementary material, further inquiries can be directed to the corresponding author.
